# Comparison of Two Components of Propolis: Caffeic Acid (CA) and Caffeic Acid Phenethyl Ester (CAPE) Induce Apoptosis and Cell Cycle Arrest of Breast Cancer Cells MDA-MB-231

**DOI:** 10.3390/molecules22091554

**Published:** 2017-09-15

**Authors:** Agata Kabała-Dzik, Anna Rzepecka-Stojko, Robert Kubina, Żaneta Jastrzębska-Stojko, Rafał Stojko, Robert Dariusz Wojtyczka, Jerzy Stojko

**Affiliations:** 1 Department of Pathology, School of Pharmacy with the Division of Laboratory Medicine in Sosnowiec, Medical University of Silesia in Katowice, Ostrogórska 30, Sosnowiec 41-200, Poland; rkubina@sum.edu.pl; 2 Department of Pharmaceutical Chemistry, School of Pharmacy with the Division of Laboratory Medicine in Sosnowiec, Medical University of Silesia in Katowice, Jagiellońska 4, Sosnowiec 41-200, Poland; annastojko@sum.edu.pl; 3 Department of Anesthesiology and Intensive Care, Prof. K. Gibiński University Clinical Center, Medical University of Silesia in Katowice, Ceglana 35, Katowice 40-514, Poland; zak@czkstojko.pl; 4 Department of Women Health, School of Health Sciences, Medical University of Silesia in Katowice, Medyków 12, Katowice 40-752, Poland; rstojko@sum.edu.pl; 5 Department and Institute of Microbiology and Virology, School of Pharmacy with the Division of Laboratory Medicine in Sosnowiec, Medical University of Silesia in Katowice, Jagiellońska 4, Sosnowiec 41-200, Poland; rwojtyczka@sum.edu.pl; 6 Department of Toxicology and Bioanalysis, School of Pharmacy with the Division of Laboratory Medicine in Sosnowiec, Medical University of Silesia in Katowice, Jagiellońska 4, Sosnowiec 41-200, Poland; jstojko@sum.edu.pl

**Keywords:** caffeic acid, caffeic acid phenethyl ester, CAPE, apotosis, cell cycle, proliferation, breast cancer, propolis

## Abstract

Studies show that caffeic acid (CA) and caffeic acid phenethyl ester (CAPE) are compounds with potent chemopreventive effects. Breast cancer is a common form of aggressive cancer among women worldwide. This study shows a comparison of CA and CAPE activity on triple-negative human caucasian breast adenocarcinoma line cells (MDA-MB-231). MDA-MB-231 cells were treated by CA and CAPE with doses of from 10 to 100 µM, for periods of 24 h and 48 h. Cytotoxicity MTT tests, apoptosis by Annexin V, and cell cycle with Dead Cell Assays were performed. Cytotoxic activity was greater for CAPE compared to CA (both incubation times, same dosage). IC_50_ values for CAPE were 27.84 µM (24 h) and 15.83 µM (48 h) and for CA > 10,000 µM (24 h) and > 1000 µM (48 h). Polyphenols induced apoptosis, while CAPE (dose dependently), induced a higher apoptotic effect. CAPE also induced cell cycle arrest in S phase (time and dose dependently), CA did it only for 50 and 100 µM. A dose dependent decline was seen for the G0/G1 phase (CAPE, 48 h), as well as elimination of phase G2/M by 100 µM of CAPE (only mild effect for CA). Comparing CA and CAPE activity on MDA-MB-231, CAPE clearly showed better activity for the same dosages and experiment times.

## 1. Introduction

Breast cancer is a common cancer and is the leading cause of cancer-related deaths among women worldwide. Studies have shown that breast cancer is a heterogeneous tumor with varying response to treatments. Radiation therapy is particularly effective in the treatment of breast cancer, but it carries the risk of normal cell damage and radioresistance of tumor cells. The development of radioresistance leads to cancer recurrence of a more aggressive phenotype in patients [1,2,3,4].

Chemopreventive agents act as cell cycle inhibitors. As a result of activation of check points, cellular stress can lead to cell cycle inhibition. G1/S phase control, prevents replication of damaged DNA, while G2/M phase control prevents segregation of damaged chromosomes into daughter cells during mitosis. Many chemopreventive factors inhibit the growth and proliferation of tumor cells by modulating the expression and/or activation of cell cycle regulatory proteins [5,6,7].

Apoptosis is a process of programmed cell death that plays a major role in the preservation of tissue homeostasis and the elimination of neoplastic cells. Chemopreventive agents can affect many effector and regulatory elements of the apoptosis process [8,9,10]. Numerous studies have shown that chemopreventive agents induce apoptosis in various types of cancer cells by affecting multiple proteins involved in programmed cell death [11,12].

The need for new compounds with effective antitumor action and high cancer cell selectivity, as well as low normal cell toxicity, highlights the need for testing a wide variety of chemically and structurally related compounds. Recently, interest in natural compounds has increased significantly as some compounds show cytotoxic, antiproliferative and proapoptotic effects essential for inhibiting cancer cell growth.

Recent studies indicate that caffeic acid (CA), and its phenethyl ester (CAPE) are compounds with potent chemopreventive effects, inter alia by cell cycle inhibition and proapoptotic action [13,14,15,16]. CAPE is a polyphenol which is a component of honeybee propolis. CAPE is known to have antiviral, anti-inflammatory, anti-cancer and antioxidant effects [17,18,19]. CAPE has shown its anticancer effects on different cancer cell lines; however, it has also exhibited differential cytotoxic activity against normal cells in the presence of cancer cells [20,21,22,23,24].

Some researchers have noted the significant role of CAPE in apoptosis and cell cycle arrest [25,26,27]. It has also been shown that CAPE can reduce expression of the mdr-1 gene and thereby increases the sensitivity of cancer cells to chemotherapy. A decrease in vascular endothelial growth factor (VEGF) concentrations inhibits angiogenesis and cancer growth [28,29,30]. Inhibition of the nuclear factor κB (NF-κB) cell signalling pathway by CAPE is also known to reflect in resistance to radiotherapy [31,32].

CA has been shown to be a factor displaying a variety of potential pharmacological effects in in vitro research as well as animal models, and it’s reported that CA has an inhibitory effect on cancer cell proliferation [33,34,35]. Nevertheless, precise reports concerning cytotoxicity and apoptotic activity of propolis components (CA and CAPE) in human cancer cells, remain inconsistent and leave the field open for further exploration. The in vitro study now reported was conducted to investigate and compare the cytotoxic effects of two phenolic substances of propolis: CA and CAPE on the viability, apoptosis, and cell cycle arrest of the MDA-MB-231 breast cancer cell line.

## 2. Results

### 2.1. Cytotoxic Effects of CA and CAPE on MDA-MB-231 Cells

**Figure 1:**
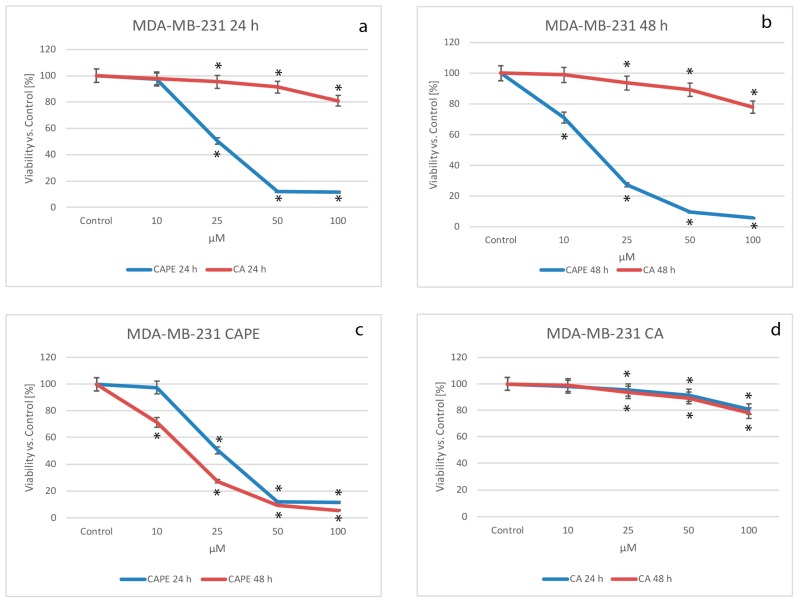
Cytotoxic effects of CAPE and CA were both tested using concentrations of from 10 to 100 µM with 24 and 48 h incubation times, on the breast cancer cell line MDA-MB- 231. Both substances caused visible dose-dependent effects. Stronger activity was observed for CAPE than CA starting with a dose of 25 µM of each compound (**a**) following a 24 h incubation time. For the 48 h experiments (**b**), all doses of CAPE resulted in a much stronger cytotoxic effect than CA using corresponding doses; (**c**) using a dose of 10 µM of CAPE, the 48 h experiment produced a more visible cytotoxic effect when compared to 24 h, and a conspicuously stronger effect for 25 µM. However, the succeeding dose increases of CAPE (50 and 100 µM) didn’t show a significant difference in viability factor, with both reaching a very low level of viability. CA cytotoxic activity showed no significant difference over time and generally, displayed low cytotoxic activity for the MDA-MB-231 cell line (**d**). Cell viability was analyzed by MTT assay. The results were presented as mean and standard deviation of three independent experiments, with 12 wells each (* *p* < 0.05; Friedman ANOVA test).

The growth inhibiting effects on a MDA-MB-231 cell line treated with CA and CAPE was assayed by MTT cell viability. Accordingly, data were normalized and expressed as % of viability over controls. Using CA for treatment of MDA-MB-231, cell viability decreased as the dose increased, dropping from 97.9% for a dose of 10 µM, to 95.4% for 25 µM and 91.4% at 50 µM and a value of 80.9% with a dose of CA 100 µM, after 24 h of incubation (Figure 1a,d). Simultaneously, comparing CAPE cytotoxic activity to CA for the MDA-MB-231 cell line (Figure 1a,c), cell viability values for a dose of 10 µM were similar to CA (97.3%), which indicates low cytotoxic effects during the experiment. However, the value was 50.3% at 25 µM, 11.9% for 50 µM and 11.6% for 100 µM.

After 48 h of incubation (Figure 1b,d), the CA cell viability had a dose-dependent effect with the following values: 99.0% for a dose of 10 µM, 93.6% for 25 µM, 89,2% for 50 µM, and finally 78.0% for 100 µM. However, if we compare the viability effect of CAPE vs. CA after 48 h of incubation (Figure 1b,c) the values were statistically different, starting with 71.2% for 10 µM of CAPE, to 27.2% for 25 µM, 9.6% for 50 µM and reaching 5.6% for 100 µM, the strongest cytotoxic effect. Therefore, CAPE demonstrated a high dose-dependent effect. Comparing CA vs CAPE, the cell viability values were statistically lower for CAPE (meaning CAPE has a higher cytotoxic effect than CA). Our results showed a dependent trend of dosages for both substances with CAPE being time dependent. It is worth noting that CAPE reached lower viability for higher doses earlier, meaning CAPE’s cytotoxic activity respectively occurs earlier.

During the experiment, the half maximal inhibitory concentration (IC_50_) was calculated, for both substances for the MDA-MB-231 breast cancer line. The results are shown in Table 1. A 50%-mortality of breast cancer cells of MDA-MB-231 were obtained with a CAPE dose of 27.84 µM for 24 h of incubation, and for 48 h–15.84 µM. For CA, the values reached more than 10,000 µM for 24 h and more than 1000 µM during the 48 h experiments. These results show that CA has lower cytotoxic activity than CAPE on MDA-MB-231 cells during both 24 and 48 h experiments.

### 2.2. Apoptotic Effects of CA and CAPE on MDA-MB-231 Cells

After exposure to CA and CAPE the MDA-MB-231 cells were stained with Annexin V bound to FITC, and analyzed by flow cytometry to assess the apoptotic cell percentages. In order to investigate the apoptotic effect of CA and CAPE, MDA-MB-231 cells were treated with both substances in the time of 24 h and 48 h, and apoptotic cells were assessed by staining with Annexin V bound to FITC and analyzed by flow cytometry to assess the apoptotic cells’ percentage. To determine whether CA/CAPE treatment results in apoptosis in MDA-MB-231 cells, we used a Muse Annexin V and Dead Cell kit to measure the changes in cell apoptosis after experimental times: 24 and 48 h.

**Figure 2:**
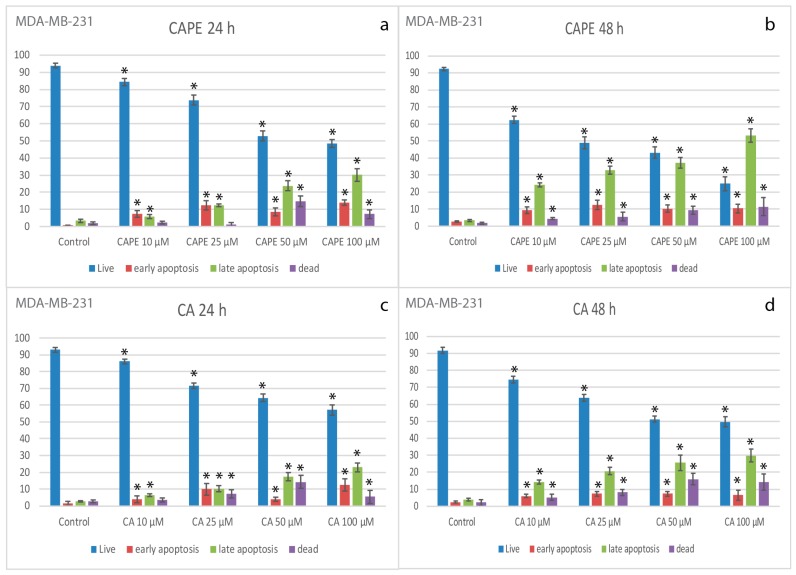
Apoptotic effect of using CA and CAPE on MDA-MB-231 cell lines using concentrations of 10, 25, 50 and 100 µM, after 24 h and 48 h incubation. For CAPE, after the 24 h period (**a**), a significant decrease in the number of live cells was observed. Early apoptosis remained stable after treatment with CAPE (24 h) and did not change significantly regardless of the dose. Late apoptosis reached up to 30.11% with the highest dosage. There was a visible dose dependent effect, when the total cells number with apoptotic phenotypes were taken into consideration. A stronger apoptotic effect was seen in the case of 48 h of incubation (**b**) with a similar situation of the number of live cells decreasing. Early apoptosis numbers were relatively stable, with late apoptosis increasing with the dosage, reaching 53.35%. In total, for CAPE at 48 h, there was a noticeable, highly dose dependent effect with apoptosis. For CA, after 24 h (**c**), a negative gradient of the number of live cells was obtained, decreasing with the dosage of CA. The apoptotic effect of CA was not as significant as for CAPE, however an increase of the number of early apoptotic cells was observed. The effect for late apoptosis was dose dependent. In total, for CA, for 24 h, with the dose dependent manner, apoptotic phenotype reached 35.47% for the 100 µM dose. (**d**) 48 h didn’t significantly change the results of live apoptosis, increased dead cells number and increase of late occurring apoptotic phenotype cells number, reached, on a dose dependent effect, were up to 29.84% for 100 µM. Vertical bars represent the standard deviation of means (SD) (*n* = 3 experiments), * *p* < 0.05 value.

We observed that both substances induced cell death through apoptosis in MDA-MB-231 cells (Figure 2). After the 24 h experiment (Figure 2a), CAPE showed a significant decrease in the number of live cells (with 93.96% for control), starting from 84.33% for 10 µM; 73.79% for 25 µM and reaching 52.9% for 50 µM and 48.52% at 100 µM. Early apoptosis (at a control value 0.65%) reached a value of 7.46% for 10 µM, with no significant change shown with a CAPE dose of up to 50 µM, however, it reached 14% at a dose of 100 µM. Late apoptosis (control value 3.38%) fluctuated up to 30.11% for the highest dose. Taking into consideration all apoptotic cell phenotypes, we observed that the number of apoptotic cells started at 13.33% for 10 µM, with the value increasing to 24.91% for 25 µM, and reaching a significant growth of 32.43% for 50 µM and 44.11% at 100 µM. A similar effect was observed of the number of live cells decreasing during the 48 h periods of incubation (Figure 2b).

However, after a 10 µM-dose treatment of CAPE with a control value of 92.24%, the number of live cells decreased by 62.23%. Then, respectively, the results were as follows: 49.04% at 25 µM, 43.18 for 50 µM, and for the highest concentration of 100 µM—24.85%. There was also a faster increase in the number of apoptotic cells. Early apoptotic cell number was quite stable with the dose increasing (control: 2.72%, but after dosage the values fluctuated between 9.26% and 12.51%), but the late apoptosis was significantly changed. With a control value of 3.32%, after a dosage of 10 µM we obtained the value of 24.15%, for 25 µM–32.85%, and a similar value of 37.29% for 50 µM, and reaching 53.35% with 100 µM of CAPE after 48 h. Taking into consideration, for all apoptotic cell phenotypes we observed a significant growth of the number of apoptotic cells (control total: 6.04%). Even after a CAPE treatment of 10 µM, we obtained a value of 33.41%, with it reaching up to 63.76% with a dose of 100 µM, for 48 h.

**Figure 3:**
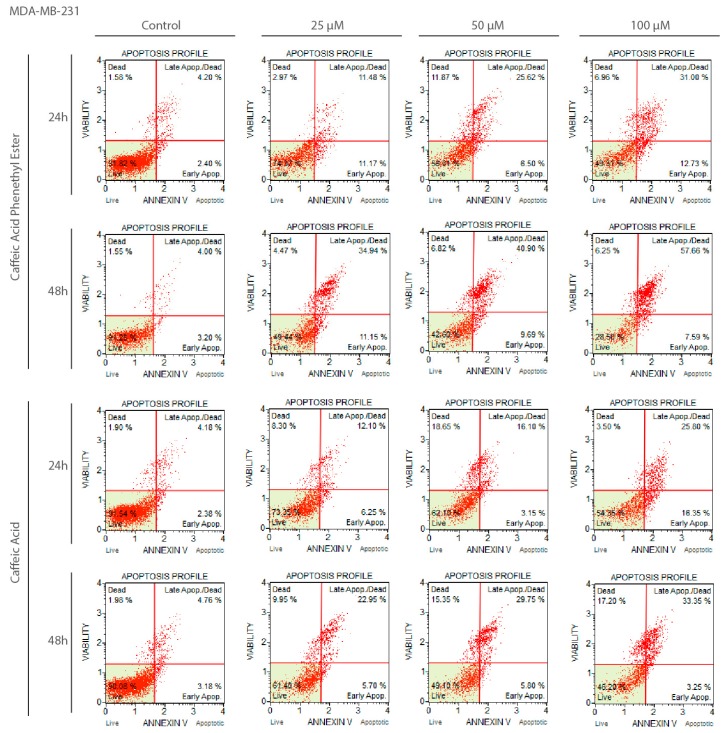
Apoptotic effect of CAPE and CA substances on MDA-MB-231 cell in the 24 and 48 h experiment (representative plots). CAPE and CA induce apoptosis in breast cancer cells of examined line. Early apoptotic cells are shown in the lower right quadrant and late apoptotic phenotype cells in the right upper quadrant of the plot. A dose-dependent effect is visible. Measured by Muse Annexin V and Dead Cell assay.

For CA, after 24 h of experiment (Figure 2c), a significant decrease in the number of live cells (control value: 93.03%) was also obtained in a dose dependent manner. Starting from 86.15% for 10 µM of CA, to 71.65% and 64.35% for 25 and 50 µM, respectively, and finally 57.17% for a dose of 100 µM. The apoptotic effect of CA was not as significant as for CAPE, however an increase of early apoptotic cells number with the treatment of this agent was observed, starting with a dose of 10 µM and reaching a value of 3.87%, then 9.84% for 25 µM. Only for 50 µM did we observe a decrease of early apoptosis (back 3.98%), but we clearly saw at the same time an abnormal growth of the number of dead cells of 14.31% (while the other dosages fluctuated from 3.56% up to 7.27%). At 100 µM, early apoptosis had the highest value of 12.47%. Late apoptosis phenotype existed during this experiment and was dependent in the dose domain, starting from 6.39% for 10 µM and reaching a maximum. Value for 100 µM of 23.01 (control: 2.77%). Total number of apoptotic cells also increased depending on dosage size, starting from 10.26% for 10 µM and reaching its extreme of 35.47% with 100 µM for the 24 h experiment. During the 48 h of incubation (Figure 2d), the CA apoptotic activity totals were somewhat similar, with the number of apoptotic cells reaching 36.35% for 100 µM, though starting from 20.25% for 10 µM. The distribution between early and late apoptosis was different than for 24 h period, fluctuating for the early phenotype from 5.91% to 7.35% (with control: 2.38%). The majority of apoptotic cells were from the late apoptosis phenotype, starting from 14.34% for 10 µM and reaching 29.84% for a CA dosage of 100 µM, having a dose dependent effect. Representative plots of apoptosis are shown in Figure 3.

### 2.3. Effects of CA and CAPE on Cell Cycle in MDA-MB-231 Cells

**Figure 4:**
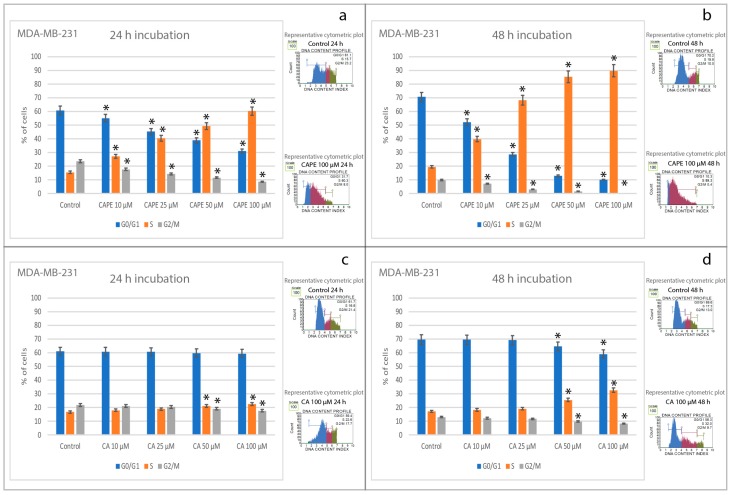
Cell cycle arrest of using CA and CAPE with concentrations of 10, 25, 50 and 100 µM after 24 and 48 h incubation on MDA-MB-231 cell line. The representative cytometric plots are shown respectively. CAPE induces cell cycle arrest in S phase, in dose and time dependent manner (**a**,**b**). Decline of G0/G1 phase and G2/M (**a**), strong dose-dependent decline G0/G1 phase for 48 h and complete elimination of phase G2/M for highest dose of CAPE (**b**). For CA’s 24 h incubation time, no significant effect in cell cycle arrest within MDA-MB-231 cells was observed (**c**). After 48 h there was a slight effect of cell cycle at phase S in dose-dependent manner, and slight but significant decline of phase G0/G1 and G2/M, but only for the highest dosages (50 and 100 µM) (**d**). Comparison of these two polyphenols evidently shows CAPE has a greater influence on cell cycle arrest induction in MDA-MB-231 than CA. Lower doses as well favorably testify to CAPE’s anticancer ability to eliminate breast cancer cells. Cells were stained with Muse Annexin V and Dead Cell kit and were subjected to flow cytometric analysis collecting 10,000 events. Vertical bars represent the standard deviation of means (SD) (*n* = 3 experiments), * *p* < 0.05 value.

In different series, MDA-MB-231 cells were treated with doses of 10, 25, 50 and 100 µM of CAPE and CA for 24 and 48 h, and cell cycle progression was determined using flow cytometry. All results are shown in Figure 4. For 24 h incubation time, CAPE induced cell cycle arrest in MDA-MB-231 cells at the S phase, changing from 15.6% at control (0 μM of CAPE) to 60.2% at 100 μM dosage. CAPE with a concurrent declined with dosage increasing in the G0/G1 phase from 60.7% at control (0 μM of CAPE) to 31.1% at 100 μM of CAPE. In parallel, the G2/M phase decreased from 23.7% at control (0 μM of CAPE) to 8.6% at 100 μM of CAPE (Figure 4a). At the same time, CA activity in cell arrest was not so spectacular. G0/G1 phase started at control (0 μM of CA) from 61.0% and didn’t change in value with an increase of the CA dose, ending with 59.4% at 100 μM of CA. The S phase changed from 16.8% at control: (0 μM of CA) up to 22.6% for 100 μM of CA dosage. In parallel, G2/M phase decreased from 22.0% at control (0 μM of CA) down to 17.7% for 100 μM of CA (Figure 4c).

For the 48 h incubation time, CAPE induced cell arrest in MDA-MB-231 cells at the S phase, strongly changing the values from 19.5% at control (0 μM of CAPE) to 89.7% at 100 μM dosage. The G2/M phase started from 9.9% at control (0 μM of CAPE) to being practically eliminated (0.2%) for the 100 μM of CAPE (Figure 4b). Respectively, for CA, a decline of phase G0/G1 was observed: starting from 69.6% at control (0 μM of CA) to 59.0% for 100 μM of CA. CA induced cell arrest in MDA-MB-231 cells at the S phase, however the cell arrest activity of CA’s results, starting from 17.2% at control (0 μM of CAPE) up to 32.6% for 100 μM of CA, were not as spectacular as CAPE. Simultaneously, the G2/M phase decreased from 13.1% (at control: 0 μM of CAPE) down to 8.4% for 100 μM of CA in the dose-dependent manner (Figure 4d).

## 3. Discussion

Despite the noticeable progress in the treatment of cancer and the introduction of new chemotherapeutics into the clinic, modern medicine is still struggling with the problem of achieving fully effective chemotherapy. There exists a strong need to better utilize existing knowledge for the development and synthesis of new potential chemotherapeutic agents that can be characterized by their efficacy, selectivity and specificity towards cancer cells. Accordingly, interest and recognition of the therapeutic potential of natural compounds has increased significantly [36,37].

Many years of research have allowed us to identify the important factors in breast cancer diagnosis, understand its development, and most importantly, the wise choice of the most effective treatment. There are biological factors, including the presence or absence of hormonal receptors in cancer cells and overexpression of the HER2 receptor or even its absence. There are cancers that do not display hormonal receptors (ER, PGR) or overexpress the HER2 receptor; these belong to a specific type breast cancer, termed triple negative (TNBC). TNBC can develop much faster than other cancers. It is therefore important to diagnose this disease and to introduce dedicated and patient-specific treatment because estrogen, progesterone and HER2 therapies are not so effective against TNBC [38,39,40,41].

Exceptionally, in breast cancer, estrogen receptor signaling plays important role in cell proliferation and vitality. MDA-MB-231 is recognized as the ideal cell line for triple-negative breast cancer research because of its minimal expression of estrogen receptor β and lack of estrogen receptors α [42].

Our research, compared cellular response of breast cancer line MDA-MB-231 breast cancer line to two constituents which normally occur in propolis: CA and its derivative—CAPE. To the best of our knowledge, this is the first time such research has been performed. Natural agents are quite popular as many “complementary” (natural) approaches to medical treatment offer novel substances apparently well suited for the treatment support of certain forms of cancer, even proving their efficacy through clinical trials [39].

Our results obtained from a flow cytometric assay clearly showed that CA and more notably CAPE induced apoptosis and growth inhibition in a time- and dose-dependent manner against breast cancer MDA-MB-231 line. When treatments with these two phenolic compounds of propolis CAPE and CA were used, the following results were observed: CAPE, cell cycle arrest in the S phase showed a reduction and even removal of the G2/M phase, while CA had a relatively weak influence on the MDA-MB-231 cell cycle (24 h was insignificant, and 48 h—slight effect) leads us to conclude that compared to CA, CAPE induces much stronger and faster cell cycle arrest. Again, to our knowledge, this is the first study comparing the effects of CAPE and CA for inducing cell cycle arrest and apoptotic effects, in MDA-MB-231 line cells has been performed.

Dead cells tests confirmed that CA/CAPE treatment diminishes the number of viable MDA-MB-231 cells. The aim of our research was to compare the cytotoxic effects of CA and CAPE on the MDA-MB-231 cell line. Our conclusion is that CA and CAPE inhibit the proliferation and reduce the viability of MDA-MB-231 cells. Taking note that, CAPE cytotoxic activity was stronger, in line of doses, and faster, in line of time than CA, respectively. These results confirm that phenolic compounds could be used as a supportive chemotherapeutic agent for certain breast cancer conditions [40].

Natural compounds, especially phenolic ones, have been demonstrated to soften the chemotherapeutic effect in tumor cells and subsequent treatment of CA and paclitaxel induce strong synergistic effects, causing antiproliferative and apoptosis of lung cancer cells, including the NF-κB pathway [41].

The results obtained showed that treatment of breast cancer cells with these two phenolic acids effectively induced apoptosis under the conditions indicated above, with a strong apoptotic effect. Chemotherapeutic agents, including propolis constituents, have been confirmed to inhibit the growth of some cancer types. Research from Chen et al. showed that CAPE acts as a radiation sensitizer in some types of cancer. Because CAPE’s activity destination is the radioresistance signaling pathway, it improves the efficiency of the radiation response [20].

The study of Omene et al. showed that CAPE inhibited MCF-7 and MDA-MB-231 cell growth, in a dose-dependent manner, and simultaneously, CAPE didn’t demonstrate a significant effect on normal cells. Additionally, CAPE firmly influenced gene and protein expression. It reduced the growth of nuclear factor NF-κB, induced cell cycle arrest, as well as apoptosis. What was significant, mdr-1 gene, which is shown responsible for the immunity of cancer cells to chemotherapy, was down-regulated by CAPE. Furthermore, CAPE in a dose dependent way, suppressed VEGF production in MDA-MB-231 cells. This phenomenon also resulted in the creation of capillary-like tubes by endothelial cells, what implicated an inhibition of angiogenesis. Their results strongly suggest that CAPE restrained MCF-7 and MDA-MB-231 cells growth through apoptosis, NF-κB modulation, carcinoma cell cycle change and arrest, as well as angiogenesis [43].

In their research, Khoram et al. noted that CAPE reduced the viability of the MDA-MB-231 and T47D cell lines. That effect was strongly correlated with the dosage and a time of experiment. They also performed a clonogenic test, followed by initial CAPE treatment (before cells were irradiated), and they found that this course widely diminished the surviving fraction of MDA-MB-231 cells at doses of 6 and 8 Gy. Comparison of the surviving fraction of both cell lines showed, that the surviving fraction of T47D was reduced at a lower dosage of radiation, compared to MDA-MB-231. Furthermore, the DNA destruction in T47D cells took longer than in MDA-MB-231 cells, when the irradiation assisted with CAPE treatment was performed. They put forth the thesis that CAPE prolonged DNA damage that could induce the radiosensitivity in the breast carcinoma cells [44].

Onori et al. also presented interesting results of their in vivo and in vitro experiments. They found that CAPE inhibited the growth of cancer cells, mainly thanks to NF-κB inhibition. This factor is exceptionally active in cholangiocarcinoma cells. They performed the CAPE treatment of nuclear extracts using dosages of 20, 40, 50 µM and confirmed that CAPE inhibited of NF-κB DNA-binding activity. Moreover, the expression of NF-κB1 (p50) and RelA (p65) was declined by CAPE. Moreover, they also observed a growth arrest among many cholangiocarcinoma cells, while was there no such activity in normal cholangiocytes. Additionally, they observed a decrease in a protein (PCNA) protection as well as in the amount of BrdU-positive cells, which were treated by CAPE (dosage 20 µM). All that resulted in a cell cycle decrease. It is worth noting that apoptosis was conjugated with a growth inhibition, as well as with the cell cycle arrest of Mz-ChA-1 cells, when treated with CAPE. Also, Bax expression was raised, while Bcl-2 was simultaneously diminished in the cells treated with CAPE. During their in vivo study, further growth of the cancer was suppressed. The tumor latency was raised twice as high with CAPE, compared to control. Sample sections demonstrated a decreased cholangiocarcinoma growth, with noticeable apoptosis. Both in vivo and in vitro experiments showed that CAPE declined the growth of cholangiocarcinoma by inducing apoptotic effect in the cancer cells [45].

Sanderson et al. showed in their interesting research, the activity of CA and its different derivatives, including CAPE, on human androgen-dependent LNCaP prostate cancer cells. They compared them with results showing that certain caffeic acid derivatives, and particularly CAPE, had potent cytotoxic effects in LNCaP cells. They compared 19 synthetic derivatives of caffeic acid and CA itself, but only three of them, including CAPE, decreased the cell viability of LNCaP cells (in a concentration-dependent way) after a 24 h exposure, and CA-related results were definitely not as promising as those obtained with CAPE, which is in line with our research. CAPE’s IC_50_ value was much lower than that of CA [46].

Research of Rosendahl et al., showed that in the breast cancer cell lines MCF-7, T47D and MDA-MB-231 (ERα-), caffeine or CA reduced human breast cancer cell growth in vitro. There was little difference in their results and ours regarding cell cycle arrest using similar doses of CA (10 and 50 µM) on MDA-MB-231. They compared CA with caffeine which induced cell cycle arrest, decreased G2/M phase and increased G0/G1 bearing in mind, a completely different dosage than CA: 1 and 5 mmol/L, respectively. With doses 10 and 50 µM of CA, no changes were observed with cell cycle on MDA-MB-231 cell line. Our research also confirmed CA as having only slight effects on MDA-MB-231 cells [47].

Jaganathan’s research also studied the use of CA on HCT 15 colon cancer cells. The dose-dependent antiproliferative effect of CA against HCT 15 colon cancer cell growth in vitro was shown using doses of 100, 200, 300, 500, 600, 800, 1000 and 2500 µM. In fact, using such large doses led him to conclude that CA could be promoted as a likely colon cancer drug candidate for chemoprevention. The IC_50_ calculations also showed this fact. Furthermore, a mitochondrial membrane potential fall was observed in the cells treated in his research. Dose- and time-dependent staining by Yo-pro-1 demonstrated the presence of apoptotic cells after CA treatment. CA can therefore be considered as a potential candidate for inducing apoptosis in colon cancer cells via ROS and a mitochondrial mediated mechanism [16].

CA was studied in our previous research. We found that caffeic acid reduced the migration rate and viability of an oral carcinoma cell line (SCC-25) when exposed to low concentrations of ethanol. There was a significant variation of MTT absorbance measurements compared to ethanol alone and a dose-dependent effect of the combination of EtOH with CA on SCC-25 cells as the values of proliferation absorbance showed. The results thus demonstrated that CA had a cytotoxic effect on the oral carcinoma cell line tested [48].

Additionally, during our earlier research we tested the apoptotic response and induction of cell cycle arrest of head and neck squamous carcinoma cells—the Detroit 562 line. Notably, the results indicated that CAPE had a greater apoptotic effect on Detroit 562 cells than did CA. Also, the findings suggest that lower doses of CA and CAPE (up to 25 μM) acting for 24 h, may not affect Detroit 562 cancer cells’ viability nor cell cycle. At a concentration of 100 μM CAPE has a mild effect on cell cycle arrest of Detroit 562. However, the cell number in the S phases and G2/M phase decreased to 31% and 18% respectively, when exposed to 100 μM of CAPE for 48 h. We still see better potential in CAPE than CA, in the respect of tested cell lines and the same dosage [13].

Interesting results were proposed by Beauregard et al. They tested CAPE and 18 of its derivatives’ activity in relation to human breast cancer MCF7 cells. They found that five of eighteen CAPE-derivatives had better apoptotic activity (through induction of caspase 3/7) than CAPE itself, on MCF-7 cells. However, they observed that there was no significant difference between CAPE-derivatives and CAPE in the inhibition of NF-κB. Interestingly, all CAPE-analogs selected in the study activated the p53 pathway in the MCF7 cells [49].

In their research, Watabe et al., confirmed that CAPE inhibits NF-κB. Moreover, they noted, that CAPE resulted in Fas death-inducing receptors being aggregated by a Fas-L-independent mechanism, in the MCF7 cells, and therefore, CAPE induced apoptosis. The death receptor clustering by CAPE resulted from both pathways, FADD/caspase-8 and JNK/p38 [50].

In our previous research, among others, we examined ethanol extract of propolis (EEP) and CAPE activity against MDA- cells MB-231 and Hs578T. The results obtained there showed strong a time- and dose-dependent trend in decreasing of cell viability, both for EEP and CAPE. The MTT assay and LDH leakage assay were used in order to measure cytotoxicity (with experimental periods of 24, 48 and 72 h). Viability values obtained in this study are coherent with the results demonstrated in the previous study [18].

Research of natural ingredients’ activity in cancer chemoprevention is widely practiced nowadays. Amatori et al., took into the consideration the activity of polyphenols against breast cancer, both in vitro and in vivo. They proposed polyphenol-rich strawberry extract (PRSE), to be used on treatment of, among others, basal-like breast cancer cell line A17 cells, which eventually, was recognized as very aggressive and invasive. They observed that PRSE induced cell cycle arrest in G1 phase. They also measured the migration rate and viability, and they observed a positive influence of PRSE on A17 cells: decreased viability in the time and dose domain. They monitored expression of 12 genes having different influence on the adhesion, migration and invasion. Also, their in vivo experiment confirmed the cancer growth inhibition, evidencing that polyphenols have broad anticancer activities [51].

Another natural chemoprevention study was performed by Pan et al. In this review, the influence of natural products, especially berries, were investigated on pancreatic ductal adenocarcinoma (PDAC). This review showed that there is a big field for chemoprevention if natural products are used. A summary of the effects and wide research, as well in vitro as clinical tests were performed, confirming the right direction of using natural agents in chemoprevention and cancer research. Also, the high intake of phytochemicals from fruits, nuts, vegetables, honey is one of the factors of cancer risk decrease [52].

## 4. Materials and Methods

### 4.1. Cell Lines and Reagents

#### 4.1.1. Breast Cancer Cell Line MDA-MB-231

For our research, we used one breast cancer line: MDA-MB-231 (human breast adenocarcinoma, TNBC, no. 92020424 SIGMA from Sigma-Aldrich, Poznań, Poland), which is a model of human triple-negative breast cancer. All manufacturer’s recommendations for preparing were carefully followed accordingly. The MDA-MB-231 cells were cultured with Leibovitz’s L-15 medium with 10% of inactivated fetal bovine serum (FBS, Sigma-Aldrich, Poznań, Poland) at 37 °C without CO_2_. The cultured cells were supplemented with antibiotics of the following concentrations: penicillin 100 U·mL^−1^, streptomycin 100 μg·mL^−1^ and fungistatic amphotericin B with a concentration of 0.25 μg·mL^−1^. We changed the medium every 48–72 h, with the passage carried out with a confluence of 80–90%.

#### 4.1.2. CA and CAPE

Caffeic acid (CA, Sigma: C0625) and caffeic acid phenethyl ester (CAPE, Sigma: C8221) were purchased from Sigma-Aldrich (Poznań, Poland) and they were all stored, collected, and used strictly according to the manufacturer’s instruction.

### 4.2. MTT TEST

Cytotoxicity of tested compounds (CA and CAPE) for use with the MDA-MB-231 line was measured by MTT (3-(4,5-dimethylthiazol-2-yl)-2-5 diphenyl tetrazolium bromide). Test of viability shows the cells’ ability to reduce MTT. After 24 and 48 h incubation of the cells with the test compounds (at concentrations of 10, 25, 50 and 100 µM), the medium was decanted and each well of MTT reagent was added at a final concentration of 1 mg/mL, and the samples then incubated for 4 h at 37 °C in 5% CO_2_. After this time, the supernatant was removed, and the water-insoluble formazan crystals were dissolved in 150 mL of DMSO. We used an ELISA plate reader (BioTek, Winooski, VT, USA) at a wavelength of 570 nm in order to read the absorbance. The same procedure we used for testing incubations of 48 h. Nutrient medium was used as a control group.

### 4.3. Muse^®^ Annexin V and Dead Cell Assay

For the analysis of flow cytometric, MDA-MB-231 cells, at an amount of 5 × 10^5^ cells per well, were plated in six well plates and allowed to stand for 24 and 48 h to obtain a logarithmic growth. The cells were incubated in a complete culture medium containing 10, 25, 50 and 100 µM of the tested compounds for 24 and 48 h. For the apoptotic assay, 1 × 10^6^ of cells in suspension were transferred to the new tube and incubated with 100 µL of Annexin V & Dead Cell reagent (Merck Millipore, Warsaw, Poland) for 20 min at room temperature. The apoptosis was determined with a Muse Cell Analyzer (Warsaw, Poland); emission max.: yellow- red- 576 nm and 680 nm, excitation max.: 532 nm).

### 4.4. Statistical Analysis

All results are expressed as means ± SD obtained from three separate experiments and performed in quadruplicates (*n* = 12). The results were performed with independent sample *t*-tests. The experimental means were compared to the means of untreated cells harvested in a parallel manner. Differences between 24 and 48 h incubated samples, were tested for significance using the one- and multiple-way Friedman ANOVA test. A *p*-value less than 0.05 were considered statistically significant.

## 5. Conclusions

With all the limitations of in vitro study, we conclude that using caffeic acid or CAPE for adjuvant therapy and/or chemoprevention is positive, but still inconclusive. Many promising results have been obtained studying selected biologically active substances isolated from bee propolis, especially polyphenols and propolis itself, but the inconsistent results make it too early to draw hard conclusions. However promising, further advanced studies are needed, particularly clinical trials, to confirm the clinical effectiveness of polyphenols on breast cancer treatment and prevention and there is still a need to know the full mechanism of CA and CAPE’s activities on cancer cells. Our comparison of CA and CAPE activity on the MDA-MB-231 clearly showed better activity of CAPE, for the same dosage and experiment time.

## Figures and Tables

**Table 1:** IC_50_ values (µM) of CA and CAPE in relation to breast cancer MDA-MB-231 for 24 h and 48 h. The obtained data demonstrates that CAPE has far bigger activity than CA on MDA-MB-231, during both the 24 and 48 h periods.

	Time of Incubation	Time of Incubation
Compounds	24 h	48 h
Caffeic acid	>10,000	>1000
Caffeic acid phenethyl ester	27.84	15.83
